# Drug-related problems in elderly patients with AECOPD and pharmaceutical intervention practice: a prospective study

**DOI:** 10.3389/fphar.2025.1596795

**Published:** 2025-06-19

**Authors:** Zengqing Ma, Hongjun Shi, Guojun Hong, Lianping Wu, Yu Lin

**Affiliations:** ^1^ Department of Pharmacy, Gaochun Hospital Affiliated to Jiangsu University, Nanjing, Jiangsu, China; ^2^ Department of Respiratory and Critical Care Medicine, Gaochun Hospital Affiliated to Jiangsu University, Nanjing, Jiangsu, China

**Keywords:** drug-related problems, chronic obstructive pulmonary disease, acute exacerbation, clinical pharmacists, hospital pharmaceutical service

## Abstract

**Background:**

Elderly patients with acute exacerbation of chronic obstructive pulmonary disease (AECOPD) face an elevated risk of drug-related problems (DRPs) owing to age-related decline in physiological reserve and multiple comorbidities. This study aims to systematically identify and categorize the DRPs of elderly patients with AECOPD, analyze their causes and risk factors, and evaluate the impact of pharmaceutical interventions on resolving DRPs.

**Methods:**

Elderly patients hospitalized with AECOPD at Gaochun Hospital Affiliated to Jiangsu University were enrolled in this prospective study from 1 January 2023 to 31 December 2024. Clinical pharmacists classified and documented DRPs using the Pharmaceutical Care Network Europe (PCNE) V9.1 Classification System, followed by targeted pharmaceutical interventions based on causes of DRPs.

**Results:**

A total of 544 AECOPD patients were included in the study. Among them, 393 DRPs were identified in 300 patients, resulting in an occurrence rate of 55.15%. “P2 Treatment safety” was the major DRPs (*n* = 177, 45.04%). Univariate and multivariate analysis showed that the number of drugs >15 (OR: 136.648, 95% CI: 38.693–482.583) and the number of comorbidities >2 (OR: 161.092, 95% CI: 43.880–591.402) were the risk factors for DRPs (P < 0.001). 472 causes were analyzed by clinical pharmacists. “C9.1 No or inappropriate outcome monitoring (including TDM)” was the most common cause of DRPs (*n* = 163, 34.53%). Clinical pharmacists proposed 435 interventions to address DRPs, of which 398 (91.49%) interventions were accepted. In the end, 294 (74.81%) DRPs were totally solved, and 18 (4.58%) DRPs were partially solved.

**Conclusion:**

The incidence of DRPs is high in elderly hospitalized patients with AECOPD in China. The number of comorbidities and prescription drugs are important predictors for DRPs. The active involvement of clinical pharmacists in the management of AECOPD patients can enhance both the safe and effective use of medications among elderly patients, as well as the overall quality of pharmaceutical care.

## 1 Introduction

Chronic obstructive pulmonary disease (COPD) is a chronic, progressive airway disease characterized by a persistent decline in lung function and airway remodeling, with a slow onset and prolonged duration (Agusti et al., 2024; Labaki and Rosenberg, 2020). Patients with COPD commonly experience symptoms such as sputum production, cough, wheezing, and dyspnea (Du et al., 2024). As the most prevalent chronic airway disease, COPD is a significant focus of prevention and treatment efforts outlined in the Healthy China 2030 Action Plan. Currently, COPD ranks as the third leading cause of death globally, accounting for over 3 million deaths annually, which represents approximately 6% of all deaths worldwide (Yu et al., 2023; Justel Enríquez et al., 2024). A health-augmented macroeconomic modeling study has projected that the economic burden of COPD will reach $4.326 trillion in 2020–2050, equivalent to an annual tax of 0.11% on global GDP (Chen et al., 2023). The World Health Organization (WHO) anticipates that the prevalence of COPD will continue to increase over the next 4 decades, driven by rising smoking rates in developing countries and aging populations in high-income nations. By 2050, it is estimated that nearly 600 million individuals worldwide will be affected by COPD (Boers et al., 2023). Furthermore, by 2060, annual mortality from COPD and its associated complications is projected to surpass 5.4 million deaths per year.

In the course of COPD, acute exacerbation of chronic obstructive pulmonary disease (AECOPD) is a dangerous stage and key event that significantly impacts patient outcomes (Qian et al., 2023). It is closely associated with deterioration of the patient’s health status, decreased quality of life, loss of workforce productivity, decline in lung function, increased medical expenses, and elevated risk of mortality (Wedzicha et al., 2017; Adhikari, 2024). Studies have shown that the 30-day readmission rate for AECOPD is approximately 22% (Bourbeau et al., 2023), while the 3-month readmission rate reaches as high as 35% (Qian et al., 2023). Furthermore, the 1-year and 5-year mortality rates for patients hospitalized due to AECOPD are 25% and 65%, respectively (Celli et al., 2023). Alarmingly, around 2%–19% of hospitalized AECOPD patients may require transfer to the intensive care unit (ICU), with in-hospital mortality rates ranging from 12% to 24% for these individuals (Qian et al., 2023). In China, the prevalence of COPD among the elderly (aged over 60) has surpassed 27% (Wang et al., 2018). These elderly patients often suffer from multiple chronic diseases, such as cardiovascular diseases, diabetes, hypertension and metabolic bone disorders, which complicate their medication regimens (Wu et al., 2025). Polypharmacy and the decline in physiological functions associated with aging make this group more prone to drug-related problems (DRPs).

The Pharmaceutical Care Network Europe (PCNE) defines a DRP as “an event or circumstance involving drug therapy that actually or potentially interferes with desired health outcomes” (Ayhan et al., 2022). DRPs can occur at any stage of drug use, covering aspects such as prescribing, dispensing, communicating medical instructions, administering medication, and drug storage. These issues are manifested specifically as adverse drug reactions, improper medication use, drug interactions, incorrect dosage or frequency of administration, and poor treatment compliance (Ali et al., 2023).

Currently, DRPs have become a significant global public health issue, resulting in an additional annual medical cost of approximately 42 billion US dollars (Chun et al., 2023). Furthermore, DRPs also reduce patients’ quality of life, increase hospitalizations, and even increase the mortality risk of patients (Kyomya et al., 2023; Chapagain et al., 2024). Multiple studies have shown that DRPs are particularly prevalent among the elderly population, with a prevalence ranging from 63.3% to 95.9% (Al-Azayzih et al., 2023). Compared to younger patients, elderly patients typically experience more DRPs (Wang et al., 2022). Specifically, the risk of DRPs in elderly patients is nearly four times that of younger patients (AOR = 3.89, 95% CI = 1.34–11.34, p = 0.013) (Kefale et al., 2022). For elderly patients with AECOPD, the complexity of the disease, the variety of medications, and changes in metabolic function contribute to the high incidence of DRPs (Bahap-Kara et al., 2024). Therefore, systematically identifying and effectively managing DRPs is of great significance for improving the treatment safety and clinical efficacy of elderly AECOPD patients.

As professionals in medication therapy management, clinical pharmacists play a crucial role in identifying, preventing, and resolving DRPs (Jimmy et al., 2023). The PCNE is currently the most widely used classification system for DRPs, consistently providing comprehensive classifications, strong professionalism, and broad applicability through its continuous updates (Chen et al., 2022; Sefera et al., 2022). By using this system, clinical pharmacists can establish a “problem identification - cause analysis - drug intervention - result evaluation” pharmaceutical intervention model, enabling them play a vital role in the identification and resolution of DRPs in elderly patients with various diseases, such as type 2 diabetes (Al-Azayzih et al., 2023), ischemic stroke (Tian et al., 2023), and surgery (Meng et al., 2021). However, only one study conducted 6 years ago evaluated the prevalence and characteristics of DRPs in COPD patients of all ages based on PCNE V8.02 (Li et al., 2019). Research on the characteristics of DRPs in elderly patients with AECOPD and the corresponding pharmacological intervention strategies remains largely unexplored. This study utilized the PCNE V9.1 classification system to identify DRPs in elderly AECOPD patients and analyzed their characteristics and risk factors. It focused on the effectiveness of individualized interventions led by clinical pharmacists in resolving DRPs and developed a standardized pharmaceutical intervention model, thereby providing a basis for improving the drug treatment management system for elderly AECOPD patients.

## 2 Methods

### 2.1 Study design, setting and subjects

We conducted a prospective, single-arm intervention study at Gaochun Hospital affiliated to Jiangsu University, which is a tertiary general hospital with 1,300 beds. The study subjects were hospitalized patients from 1 January 2023 to 31 December 2024. As this was an exploratory study, sample sizes were not calculated.

Inclusion criteria: (1) Patients diagnosed with AECOPD; (2) Age >60 years old; (3) Good compliance of patients and willingness to cooperate with follow-up; (4) Voluntary participation.

Exclusion criteria: (1) Incomplete clinical data of the patient with key information missing; (2) Severe or terminal diseases; (3) Mental disorders or severe cognitive dysfunction.

### 2.2 Data collection

Two clinical pharmacists (Z Ma and H Shi) and a pulmonologist (Y Lin) collected patient information from the electronic hospital information system (eHIS) and patient interviews, including age, gender, smoking history, laboratory test results, comorbidities, length of stay (LOS), and medication information. The two clinical pharmacists (Z Ma and H Shi) also recorded the classification, causes, and interventions of DRPs, and documented the acceptance and outcomes of the interventions 48 h after they were proposed.

### 2.3 Identification and classification of DRPs

At the outset of the study, all clinical pharmacists underwent standardized training on the PCNE V9.1 classification system, which included rule interpretation and case discussion to ensure a consistent understanding of the classification system among them. During the study, two clinical pharmacists with over 5 years of experience (Z Ma and H Shi) independently reviewed the patients’ medication records to identify potential DRPs based on the latest scientific references, including clinical guidelines, drug instructions, and the UpToDate website. These medication records included bronchodilators, antibiotics, corticosteroids, cough suppressants, antihypoxic drugs, expectorants, acid suppressants, antihypertensive drugs, and hypoglycemic drugs. Subsequently, the identified DRPs were classified independently by the two pharmacists according to the PCNE V9.1 classification system. When there was a dispute in the classification of DRPs, a third clinical pharmacist with over 10 years of experience (L Wu) was consulted, and a consensus was reached through discussion grounded in relevant literature and clinical evidence. Once the DRPs were identified, the clinical pharmacists provided interventions immediately. The PCNE V9.1 classification system consists of five sections: problems (3 primary domains), causes (9 primary domains), interventions (5 primary domains), acceptances (3 primary domains), and outcomes (4 primary domains). A single problem may be caused by multiple factors, and various interventions may be implemented. However, ultimately, there is only one outcome.

### 2.4 Statistical analysis

According to the data distribution, categorical variables were expressed as frequencies and percentages, and continuous variables were expressed as mean ± Standard deviation (M ± SD). Univariate and multivariate logistic regression analyses were used to determine the potential risk factors of DRPs. Network diagram between causes and DRPs was plotted by https://www.bioinformatics.com.cn, an online platform for data analysis and visualization. Statistical analysis was conducted using SPSS Statistics 22.0 software, and *P* < 0.05 was considered statistically significant.

### 2.5 Ethical approval

This study was approved by the Ethics Committee of Gaochun Hospital Affiliated to Jiangsu University (Approval Number: 2023-098-01), and was conducted in accordance with the Declaration of Helsinki.

## 3 Results

### 3.1 Patient enrollment

**FIGURE 1:**
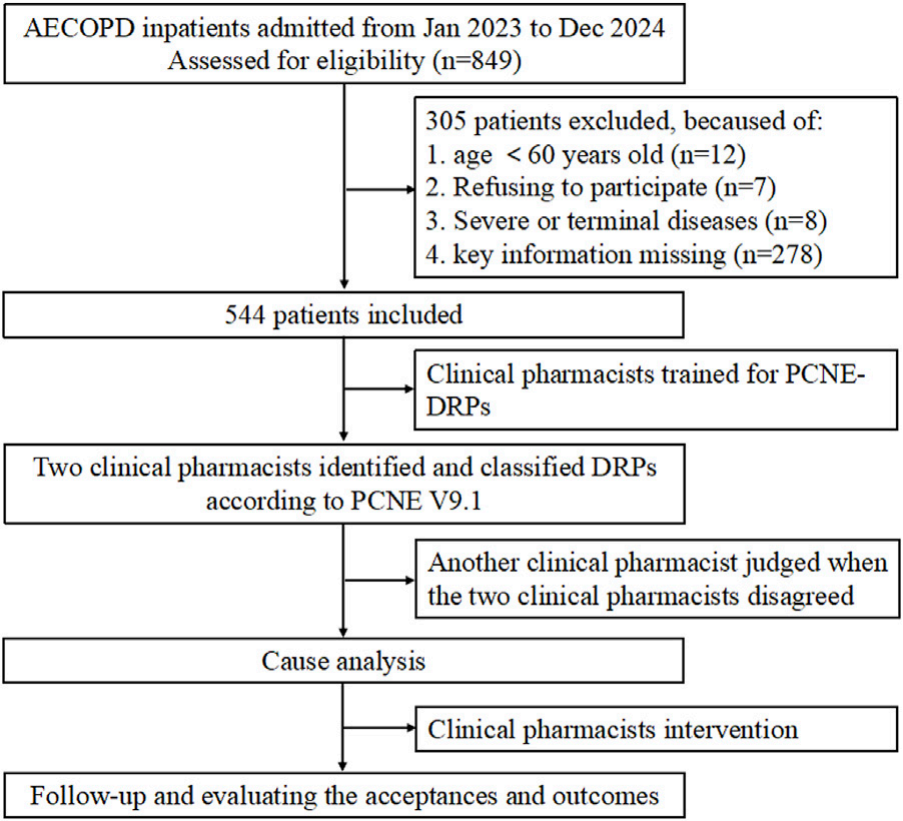
Flowchart of the study.

During the 2-year study period, a total of 849 patients were hospitalized due to AECOPD. Twelve patients were excluded because they were under 60 years old, as individuals aged 60 years or older are legally defined as “elderly” in China according to the Law of the People’s Republic of China on the Protection of the Rights and Interests of the Elderly (2018 Amendment). Seven patients were excluded for refusing to participate in the study, and eight patients met exclusion criteria due to the presence of severe or terminal illnesses. Additionally, 278 patients were excluded for incomplete clinical data, including missing results of pulmonary function tests and routine blood tests during hospitalization, which precluded their inclusion in the statistical analysis of this study. Ultimately, 544 patients were included (Figure 1).

### 3.2 Patient characteristics

The majority of patients participating in this study were male (*n* = 474, 87.13%), while female patients accounted for 70 individuals (12.87%). The mean age of the patients was 75.18 years, and 83.27% of them had a history of smoking. Regarding the patients’ pulmonary function, the average FEV_1_% was 39.31%, with over 88% of the patients having severe or very severe COPD. Specifically, 47.43% of patients were categorized as having severe COPD (GOLD grade 3), and 31.43% were classified as having very severe COPD (GOLD grade 4). The average LOS for the patients exceeded 1 week, at 8.80 days. During the hospitalization period, each patient used an average of 15.01 drugs, with bronchodilators, glucocorticoids, expectorants, antibiotics, and antitussives constituting the five most frequently administered drug categories. Concurrently, the study subjects exhibited a mean of 3.32 comorbidities per individual, with hypertension (40.26%, *n* = 219), hyperlipidemia (13.79%, *n* = 75), and chronic gastritis (11.21%, *n* = 61) being the predominant concurrent diagnoses. Detailed characteristics of the AECOPD patients are presented in Table 1.

**TABLE 1:** Characteristics of AECOPD patients.

Characteristics	Value
Gender	
Male	474 (87.13%)
Female	70 (12.87%)
Age	75.69 ± 5.91
Smoking history	453 (83.27%)
FEV_1_%	39.31% ± 15.15%
GOLD grade	
Grade 1	8 (1.47%)
Grade 2	107 (19.67%)
Grade 3	258 (47.43%)
Grade 4	171 (31.43%)
Length of stay	8.80 ± 3.78
Number of drugs	15.01 ± 5.45
Comorbidity	3.32 ± 2.51
Hypertension	219 (40.26%)
Hyperlipemia	75 (13.79%)
Chronic gastritis	61 (11.21%)
COVID-19	57 (10.48%)
Coronary heart disease	55 (10.11%)
Diabetes	49 (9.01%)
Chronic kidney disease	47 (8.64%)

### 3.3 Prevalence and distribution of DRPs

**FIGURE 2:**
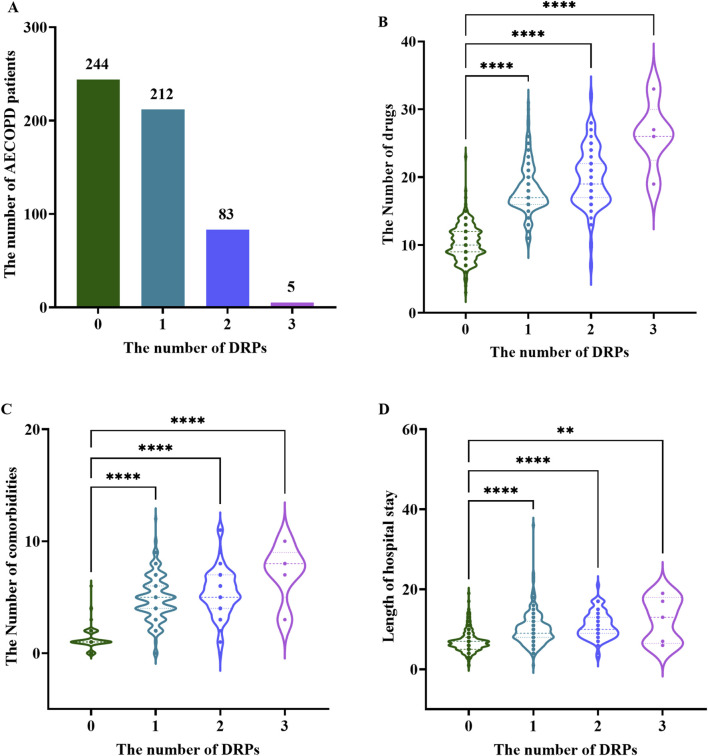
Distribution of DRPs in elderly patients with AECOPD **(A)** Number of DRPs per patient; **(B)** Violin plot of the number of DRPs and drugs; **(C)** Violin plot of the number of DRPs and comorbidities; **(D)** Violin plot of the number of DRPs and LOS. **: P<0.01; ****: P<0.0001.

Of the 544 patients included in the study, 300 (55.15%) experienced at least one drug-related problem (DRP), whereas 244 (44.85%) had no DRPs identified during hospitalization. Further stratification demonstrated that 212 patients (38.97%) experienced a single DRP, 83 (15.26%) encountered two DRPs, and a clinically noteworthy subset of five patients (0.92%) experienced three DRPs (Figure 2A). When conducting a correlation analysis for all patients, the number of DRPs was positively correlated with the number of drugs (Figure 2B), the number of comorbidities (Figure 2C), and the LOS (Figure 2D), with statistical significance (all *P* < 0.05).

### 3.4 Types of DRPs

In this study, clinical pharmacists identified a total of 393 DRPs (Table 2). According to the PCNE V9.1 classification system, “Treatment safety” emerged as the most prevalent types (*n* = 177, 45.04%), followed by “Treatment effectiveness” (*n* = 111, 28.24%) and “Others” (*n* = 105, 26.72%). Upon further classification, “P2.1 Treatment safety” was the subtype that occurred most frequently (*n* = 177, 45.04%), followed by “P3.2 Unnecessary drug treatment” (*n* = 91, 23.16%) and “P1.2 Effect of drug treatment not optimal” (*n* = 86, 21.88%).

**TABLE 2:** Types of DRPs (*n* = 393).

Primary domain	Code and problem	Frequency
P1 Treatment effectiveness	P1.1 No effect of drug treatment	23 (5.85%)
	P1.2 Effect of drug treatment not optimal	86 (21.88%)
	P1.3 Untreated symptoms or indication	2 (0.51%)
P2 Treatment safety	P2.1 Treatment safety	177 (45.04%)
P3 Others	P3.1 Problem with cost-effectiveness of the treatment	12 (3.05%)
	P3.2 Unnecessary drug treatment	91 (23.16%)
	P3.3 Unclear problem/complaint	2 (0.51%)

### 3.5 Causes of DRPs

A total of 472 causes were identified for the 393 DRPs mentioned above. “C1 Drug selection” was the main cause leading to DRPs (*n* = 218, 46.19%), followed by “C9 other” (*n* = 163, 34.53%) and “C7 patient related” (*n* = 27, 5.72%). Subgroup analysis revealed that “C9.1 No or inappropriate outcome monitoring (including TDM)” was the cause of C9 (*n* = 163, 34.53%), mainly due to the failure to conduct timely blood drug concentration monitoring when using theophylline drugs. “C1.1 Inappropriate drug according to guidelines/formulary” was the main indication of C1 (*n* = 128, 27.12%), mainly referring to the inappropriate selection of bronchodilators and antibiotics. “C1.2 No indication for drug” was another cause of C1 (*n* = 63, 13.352%), mainly due to the irrational use of acid-suppressing drugs (such as proton pump inhibitors and H2 receptor antagonists). The details are shown in Table 3.

**TABLE 3:** Causes of drug-related problems (*n* = 472).

Primary domain	Code and cause	Frequency
C1 Drug selection	C1.1 Inappropriate drug according to guidelines/formulary	128 (27.12%)
	C1.2 No indication for drug	63 (13.35%)
	C1.3 Inappropriate combination of drugs, or drugs and herbal medications, or drug and dietary supplements	3 (0.64%)
	C1.4 Inappropriate duplication of therapeutic group or active ingredient	10 (2.12%)
	C1.5 No or incomplete drug treatment in spite of existing indication	6 (1.27%)
	C1.6 Too many different drugs/active ingredients prescribed for indication	8 (1.69%)
C2-Drug form	C2.1 Inappropriate drug form/formulation (for this patient)	5 (1.06%)
C3 Dose selection	C3.2 Drug dose of a single active ingredient too high	8 (1.69%)
	C3.4 Dosage regimen too frequent	1 (0.21%)
	C3.5 Dose timing instructions wrong, unclear or missing	6 (1.27%)
C4 Treatment duration	C4.1 Duration of treatment too short	1 (0.21%)
	C4.2 Duration of treatment too long	16 (3.39%)
C5 Dispensing	C5.1 Prescribed drug not available	3 (0.64%)
	C5.3 Wrong drug, strength or dosage advised (OTC)	1 (0.21%)
	C5.4 Wrong drug or strength dispensed	2 (0.42%)
C6 Drug use process	C6.1 Inappropriate timing of administration or dosing intervals by a health professional	10 (2.12%)
	C6.3 Drug over- administered by a health professional	2 (0.42%)
	C6.5 Wrong drug administered by a health professional	2 (0.42%)
	C6.6 Drug administered via wrong route by a health professional	7 (1.48%)
C7 patient related	C7.1 Patient uses/takes less drug than prescribed or does not take the drug at all	6 (1.27%)
	C7.5 Patient takes food that interacts	2 (0.42%)
	C7.6 Patient stores drug inappropriately	1 (0.21%)
	C7.7 Inappropriate timing or dosing intervals	9 (1.91%)
	C7.8 Patient administers/uses the drug in a wrong way	4 (0.85%)
	C7.9 Patient unable to use drug/form as directed	3 (0.64%)
	C7.10 Patient unable to understand instructions properly	2 (0.42%)
C9 other	C9.1 No or inappropriate outcome monitoring (including TDM)	163 (34.53%)

### 3.6 Relationship between the types and causes of DRPs

**FIGURE 3:**
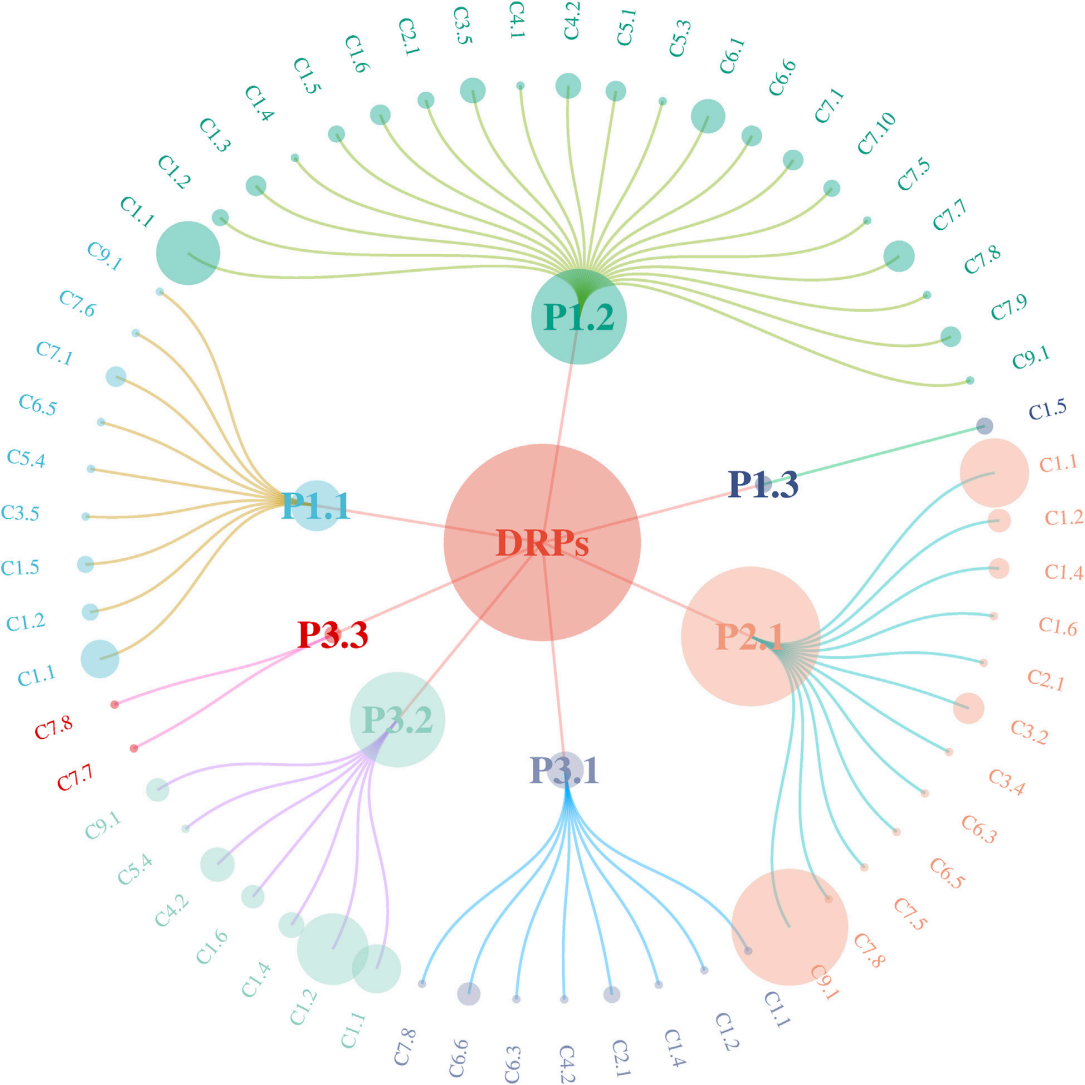
Network diagram between the types and causes of DRPs.

Figure 3 is a network diagram that illustrates the relationships between DRPs and their underlying causes. Lines connect each DRP to the cause that leads to it. The size of the bubbles represents the frequency of occurrence, with larger bubbles indicating a higher frequency. The primary cause of “P1.1 No effect of drug treatment” was identified as “C1.1 Inappropriate drug according to guidelines/formulary” (*n* = 13). There were 21 subtypes of causes (*n* = 102) identified for “P1.2 Effect of drug treatment not optimal,” among which “C1.1 Inappropriate drug according to guidelines/formulary” (*n* = 42) was the predominant cause. For “P2.1 Treatment safety,” the main causes were “C9.1 No or inappropriate outcome monitoring (including TDM)” (*n* = 157) and “C1.1 Inappropriate drug according to guidelines/formulary” (*n* = 13). Additionally, “C1.2 No indication for drug” (*n* = 54) and “C1.1 Inappropriate drug according to guidelines/formulary” (*n* = 23) were significant reasons for “P3.2 Unnecessary drug treatment.”

### 3.7 Potential risk factors of DRPs

**FIGURE 4:**
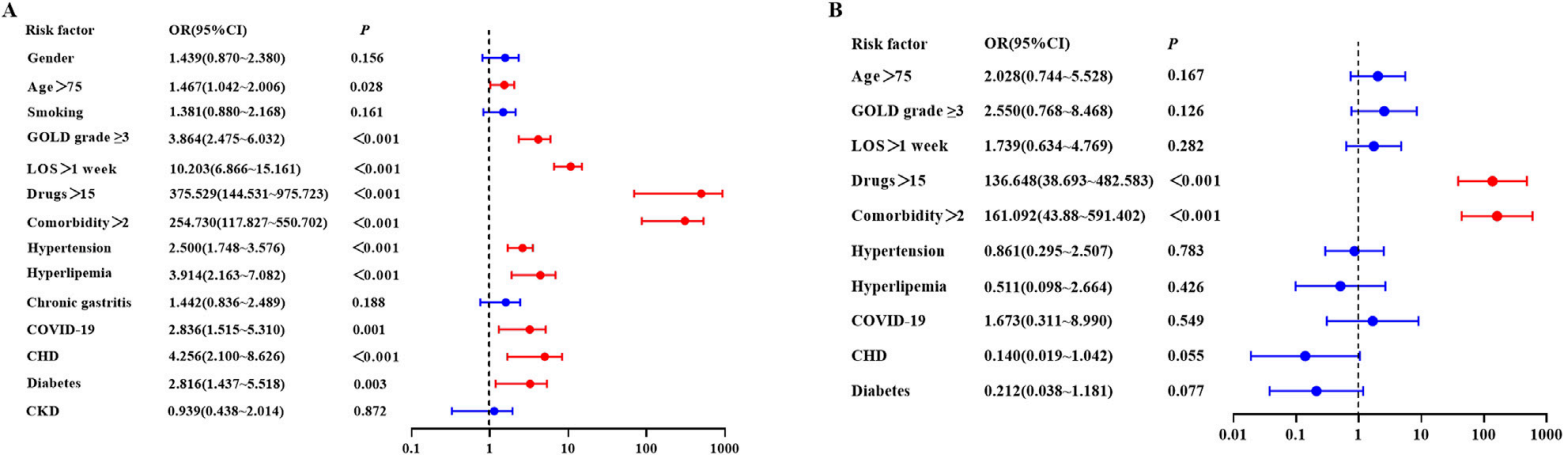
Risk factors for DRP using logistic regression **(A)** Univariate logistic regression; **(B)** Multivariate logistic regression.

Univariate logistic regression analysis (Figure 4A) revealed that age (OR: 1.467, 95% CI: 1.042–2.006, p = 0.028), GOLD grade (OR: 3.864, 95% CI: 2.475–6.032, p < 0.001), LOS (OR: 10.203, 95% CI: 6.866–15.161, p < 0.001), the number of medications (OR: 375.529, 95% CI: 144.531–975.723, p < 0.001), the number of comorbidities (OR: 254.730, 95% CI: 117.827–550.702, p < 0.001), hypertension (OR: 2.500, 95% CI: 1.748–3.576, p < 0.001), hyperlipidemia (OR: 3.914, 95% CI: 2.163–7.082, p < 0.001), COVID-19 (OR: 2.836, 95% CI: 1.515–5.310, p = 0.001), CHD (OR: 4.256, 95% CI: 2.100–8.626, p < 0.001), and diabetes (OR: 2.816, 95% CI: 1.437–5.518, p = 0.003) were identified as independent risk factors for the occurrence of DRPs. In contrast, gender, smoking, chronic gastritis, and CKD were not significant predictors of DRPs (P > 0.05). Multivariate logistic regression analysis (Figure 4B) indicated that the number of drugs and comorbidities were contributing factors to the occurrence of DRPs. Patients taking more than 15 drugs had a 136.648-fold increased likelihood of experiencing DRPs (95% CI: 38.693–482.583; P < 0.001). Additionally, patients with more than two comorbidities, the possibility of experiencing DRPs was 161.092 times (95% CI: 43.88–591.402, p < 0.001).

### 3.8 Clinical pharmacist interventions

For the 393 identified DRPs, clinical pharmacists proposed a total of 435 interventions (Table 4). 67.36% of the interventions were provided at the “prescriber level” (*n* = 293), mainly including “I1.3 Intervention proposed to prescriber” (*n* = 255, 58.62%) and “I1.4 Intervention discussed with prescriber” (*n* = 25, 5.75%). The second largest proportion of interventions was at the “drug level” (*n* = 94, 21.61%), mainly including “I3.5 Drug paused or stopped” (*n* = 37, 8.51%) and “I3.1 Drug changed to…” (*n* = 19, 4.37%).

**TABLE 4:** Interventions of drug-related problems (*n* = 435).

Primary domain	Code and intervention	Frequency (%)
I0 No intervention	I0.1 No intervention	7 (1.61%)
I1 At prescriber level	I1.1 Prescriber informed only	10 (2.30%)
	I1.2 Prescriber asked for information	3 (0.69%)
	I1.3 Intervention proposed to prescriber	255 (58.62%)
	I1.4 Intervention discussed with prescriber	25 (5.75%)
I2 At patient level	I2.1 Patient (drug) counselling	7 (1.61%)
	I2.2 Written information provided (only)	5 (1.15%)
	I2.3 Intervention proposed to prescriber	5 (1.15%)
	I2.4 Spoken to family member/caregiver	16 (3.68%)
I3 At drug level	I3.1 Drug changed to …	19 (4.37%)
	I3.2 Dosage changed to …	9 (2.07%)
	I3.3 Formulation changed to …	2 (0.46%)
	I3.4 Instructions for use changed to …	15 (3.45%)
	I3.5 Drug paused or stopped	37 (8.51%)
	I3.6 Drug started	12 (2.76%)
I4 Other intervention or activity	I4.1 Other intervention (specify)	3 (0.69%)
	I4.2 Side effect reported to authorities	5 (1.15%)

### 3.9 Acceptance of interventions and the outcomes of DRPs

As shown in Table 5, the overall acceptance rate was 91.49% (*n* = 398). Among these interventions, 85.06% were fully implemented (*n* = 370), 4.14% were partially implemented (*n* = 18), 1.38% were not implemented (*n* = 6), and 0.92% were unknown (*n* = 4). Additionally, 5.52% of the interventions were not accepted (*n* = 24), with the main reason being disagreement (*n* = 14, 3.22%).

**TABLE 5:** Acceptance of interventions and the outcomes of DRPs.

Primary domain	Code and detailed classification	Frequency
Acceptance		
A1 Intervention accepted	A1.1 Intervention accepted and fully implemented	370 (85.06%)
	A1.2 Intervention accepted, partially implemented	18 (4.14%)
	A1.3 Intervention accepted but not implemented	6 (1.38%)
	A1.4 Intervention accepted, implementation unknown	4 (0.92%)
A2 Intervention not accepted	A2.1 Intervention not accepted: not feasible	5 (1.15%)
	A2.2 Intervention not accepted: no agreement	14 (3.22%)
	A2.3 Intervention not accepted: other reason (specify)	4 (0.92%)
	A2.4 Intervention not accepted: unknown reason	1 (0.23%)
A3 Other	A3.1 Intervention proposed, acceptance unknown	4 (0.92%)
	A3.2 Intervention not proposed	9 (2.07%)
Outcome		
O0 Not known	O0.1 Problem status unknown	13 (3.31%)
O1 Solved	O1.1 Problem totally solved	294 (74.81%)
O2 Partially solved	O2.1 Problem partially solved	18 (4.58%)
O3 Not solved	O3.1 Problem not solved, lack of cooperation of patient	33 (8.40%)
	O3.2 Problem not solved, lack of cooperation of prescriber	9 (2.29%)
	O3.3 Problem not solved, intervention not effective	21 (5.34%)
	O3.4 No need or possibility to solve problem	5 (1.27%)

Under the intervention of clinical pharmacists, 74.81% of DRPs were totally solved (*n* = 294), and 4.58% of DRPs were partially solved (*n* = 18). 17.30% of DRPs remained unsolved, with the reasons being lack of patient cooperation (*n* = 33, 8.40%), lack of prescriber cooperation (*n* = 9, 2.29%), ineffective intervention (*n* = 21, 5.34%), and no need or possibility to solve the problem (*n* = 5, 1.27%). Additionally, the status of 13 DRPs was unknown.

## 4 Discussion

The incidence rate of COPD in China is showing an upward trend, with approximately 100 million patients, among whom about 27 million are elderly (Wang et al., 2018). Currently, most studies led by pharmacists on COPD primarily focus on patients in the stable phase of the disease. Previous studies have demonstrated that pharmacist interventions can significantly improve the inhalation technique of patients with stable COPD, enhance their medication adherence, and reduce their COPD Assessment Test (CAT) scores, thereby positively impacting COPD treatment (Bhattarai et al., 2024; Tam et al., 2024). However, there is a notable gap in research addressing the DRPs of elderly hospitalized patients experiencing acute exacerbations, which represents a critical area for further exploration and intervention. To our knowledge, this study is the first to investigate DRPs in hospitalized elderly patients with AECOPD in China to assess the value of pharmacists in the clinical practice.

In this study, pharmacists investigated the occurrence, types, causes and risk factors of DRPs in elderly patients with AECOPD and conducted interventions. The results showed that during hospitalization, the incidence of DRPs in elderly patients with AECOPD was 55.15%, with an average of 0.72 DRPs per patient. In contrast, a prospective descriptive study conducted in the neonatal intensive care unit (NICU) reported a much higher incidence of DRPs at 89.6%, with an average of 5.45 DRPs per patient (Ahmed et al., 2024). Additionally, in 2022, a study in Pakistan showed that patients with chronic kidney disease had a high prevalence of DRPs, with an occurrence rate of 100% and an average of 2.90 DRPs per patient (Alam et al., 2024). Compared to these studies, the prevalence and frequency of DRPs in our study were lower. This difference could be attributed to the presence of an intelligent prescription review system in our hospital, designed to promote clinically rational drug use. Through pre-prescription evaluations, this system effectively reduced the number of irrational prescriptions, thereby lowering the likelihood of DRPs. Further research indicated that the number of DRPs was positively correlated with the number of drugs, the number of comorbidities, and the length of hospital stay. This implied that as the number of treatment drugs increased, the number of comorbidities rose, and the duration of hospitalization extended, patients were likely to encounter a corresponding increase in DRPs (Larasati et al., 2024). These findings suggested that in the context of polypharmacy, along with the complexity of underlying diseases and the prolongation of hospitalization periods, patients might face risks such as multi-system pathophysiological changes and inadequate compensation of hepatic and renal metabolic functions. These factors made drug selection, dose adjustment, and treatment monitoring more challenging.

Treatment safety was the most common type of DRPs in this study, accounting for 45.04% of all DRPs, mainly represented by the possibility of potential adverse drug events. Treatment effectiveness was the second most common type of DRPs, mainly manifested as poor or no therapeutic effect. “C9.1 No or inappropriate outcome monitoring (including TDM)” was the most common cause of DRPs in this study, which was also the primary reason for treatment safety. The main manifestation was the failure to monitor blood drug concentration during the use of theophylline, which was a risk that cannot be ignored for the safe treatment of patients. Theophylline is methylxanthine derivatives with a low therapeutic index, a narrow safety range, and significant individual differences, and their therapeutic effects are mostly realized only at doses close to toxic levels (Ram et al., 2002). Moreover, some patients were concurrently using cimetidine, which could inhibit CYP450 enzymes, leading to reduced hepatic metabolism and delayed clearance of theophylline (Scurek and Brat, 2024). This could cause increased blood concentrations and potentially result in toxicity. Moreover, clearance of the theophylline declines with age and many other physiological variables and drugs modify theophylline metabolism. Therefore, therapeutic drug monitoring (TDM) of theophylline is particularly necessary for elderly patients. “C1.1 Inappropriate drug according to guidelines/formulary” was the second most common cause of DRPs, and the incorrect use of theophylline was the main factor contributing to it. In fact, in some hospitals in China, intravenous methylxanthines was still used as first-line treatment for COPD. However, as non-selective PDE4 inhibitors, theophylline had a relatively small bronchodilator effect in the treatment of COPD. The use of theophylline was not recommended unless other bronchodilators were unavailable or unaffordable (Agusti et al., 2024).

Based on the types and causes of the identified DRPs, clinical pharmacists conducted a total of 435 interventions. In this study, clinical pharmacists prioritized intervention at the prescriber level in the majority of cases (67.36%), as prescribers played a dominant role in patients’ treatment decisions. Subsequently, interventions at the drug level accounted for 21.61%, while interventions at the patient level represented only 7.59%. In 2021, a multicenter prospective observational study was conducted in Ethiopia to evaluate the effectiveness of clinical pharmacists’ interventions in addressing DRPs among elderly patients in medical wards. Similar to our findings, 52.05% of interventions in that study occurred at the prescriber level, 37.78% at the drug level, and 10.18% at the patient level (Dagnew et al., 2022). Meanwhile, a study conducted in Palestine showed that the proportion of clinical pharmacists’ interventions at the prescriber level was the highest (47.72%), followed by drug level (39.21%) and patient level (13.07%) (Elhabil et al., 2022). In comparison, the proportion of interventions at the patient level in our study was lower, suggesting that there might be a lack of communication between clinical pharmacists and patients.

In our study, 91.49% of the interventions were accepted, with 74.81% of DRPs totally solved and 4.58% partially solved. These findings are consistent with those from a prospective study conducted among patients with respiratory diseases in China, which reported a 96.25% acceptance rate of clinical pharmacists’ interventions (Zhu et al., 2019). Furthermore, in that study, 81.59% of DRPs were totally solved, and 10.24% were partially solved. These outcomes indicate that clinical pharmacists are important members of multidisciplinary teams and play an irreplaceable role in delivering high-quality drug therapy to patients.

The presence of more than two comorbidities is one of the predictive factors for the occurrence of DRPs. As the number of comorbidities an individual has increases, their health condition tends to deteriorate, drug metabolism becomes more complex, and treatment plans become more complicated, which increases the likelihood of DRPs. Additionally, the number of prescribed drugs >15 is an independent predictor of DRPs. This may be due to the greater likelihood of drug-drug interactions and drug-disease interactions that arise with an increased number of prescribed medications, leading to more DRPs.

This study has several limitations. First, it was a single-center study, which limited the generalizability of our findings. Multi-center participation is necessary to validate the pharmaceutical Intervention model in elderly patients with AECOPD. Second, this study focused primarily on identifying and resolving DRPs during hospitalization. We did not conduct long-term follow-up to assess the sustained clinical outcomes, such as readmission rates, and their relationship with the outcomes of DRPs. Third, we did not evaluate the economic implications of clinical pharmacist interventions for patients in this study.

In the future, we plan to conduct multi-center studies with long-term follow-up of elderly patients with AECOPD to comprehensively analyze the impact of resolving DRPs on treatment outcomes, prognosis, and economic aspects, thereby further enriching and enhancing the clinical value of pharmaceutical care in COPD management.

## 5 Conclusion

This study showed that DRPs were common among elderly patients hospitalized due to AECOPD. Comorbidities and the use of multiple prescribed drugs were risk factors for DRPs. Clinical pharmacists could effectively identify DRPs during daily ward rounds and efficiently solve them through communication and cooperation with physicians and education for patients.

## Data Availability

The original contributions presented in the study are included in the article/supplementary material, further inquiries can be directed to the corresponding authors.
